# COVID-19 Crisis and the Necessity for the Quick Response to the Rohingya Refugees in Bangladesh

**DOI:** 10.1017/dmp.2021.76

**Published:** 2021-03-12

**Authors:** Nafiul Mehedi, Md. Ismail Hossain

**Affiliations:** Department of Social Work, Shahjalal University of Science and Technology, Sylhet, Bangladesh

Like many other countries, Bangladesh documented its first coronavirus disease 2019 (COVID-19) case on March 8, 2020,^1^ and since then, the rate of COVID-19 positive patients is increasing swiftly. One of the least highlighted issues associated with the pandemic is the spread of coronavirus in the Rohingya refugee camps located in Cox’s Bazar district under the Chittagong division of Bangladesh, which accommodates 1.3 million Rohingya people.^2^ The refugee camps, with a huge population, are severely susceptible to the COVID-19 pandemic. The first Rohingya refuge was tested COVID-19 positive on May 14, 2020; thenceforth, a total of 367 refugees were infected with the virus. Subsequently, the first death of a Rohingya refugee was reported on May 31, 2020, and approximately 15,000 refugees were placed in quarantine as the number of cases increased at that time.^3,4^ Several aid groups and the government prepared isolation and treatment facilities, which are inadequate, as there are only 400 available beds in isolation facilities for the refugees and the host communities. More than 4100 hospital beds are required to treat critical patients in the area.^5^

At the onset of the outbreak, the supply of face masks was insufficient to cover a large number of refugees. Yet, some non-governmental organizations (NGOs) initiated training the refugees to produce re-usable masks.^6^ In addition, testing such a large number of refugees is a challenge for the respective authority. Currently, the collected samples are being tested in the World Health Organization (WHO)-supported Institute of Epidemiology, Disease Control and Research (IEDCR) Field Laboratory (PCR laboratory) in Cox’s Bazar district. Meanwhile, a reduction in the number of tests conducted among the refugees was noted in week 53, from 914 to 706 tests.^7^

Thousands of development workers from 98 NGOs,^8^ government officials, and security forces work there, and they are continuing their duties amid the COVID-19 outbreak. Hence, the development workers would be the possible vectors for the outbreak of the disease in the camps for their daily entry-exit.^9^ Moreover, the radical increase of COVID-19 positive cases in the Rohingya camps is a matter of serious concern for the local community and the aggregate health sector of Bangladesh. Hereafter, the government and the NGOs should focus more earnestly on the Rohingya camps to control the spread as it may lead to a disaster in the health sector of the country. Some potential recommendations to control the spread in the Rohingya camps are as follows:

- Daily COVID-19 symptoms screening for the workforce engaged in the camps should be strictly performed.
- The respected authorities should take more rapid responses to test suspects and provide medical care to the confirmed patients, including the refugees, governmental organizations (GOs) and NGO workers, security forces, and the local community people.
- The GOs and NGOs should train and fund the refugees to contribute to the non-pharmaceutical interventions (NPIs), such as producing face masks
- The refugees and the workers in the camps should be advised to follow the NPIs, such as maintaining social distancing and using face masks.
- Finally, sufficient isolation centers and hospital beds are needed to avoid a potential COVID-19 related health hazard. In this regard, the GOs and NGOs can build temporary tents in the open spaces, away from the local community.
